# Clinical Profiles and Surgical Outcome of Hypospadias Repair at a Teaching Hospital in Ethiopia

**DOI:** 10.4314/ejhs.v32i3.18

**Published:** 2022-05

**Authors:** Maru Gama, Birhan Abitew, Kirubel Abebe

**Affiliations:** 1 Pediatric surgery unit, Department of surgery, St. Paul's hospital millennium medical college, Addis Ababa, Ethiopia; 2 Department of surgery, St. Paul's hospital millennium medical college, Addis Ababa, Ethiopia

**Keywords:** Hypospadias, Repair, Complication, Ethiopia

## Abstract

**Background:**

Hypospadias repair is one of the commonest and challenging surgery done in pediatric age groups. This study was conducted to assess clinical profiles and surgical outcomes of hypospadias repair.

**Methods:**

A retrospective analysis of pediatric hypospadias repairs at St. Paul's hospital millennium medical college from September 2015 to August 2019 was conducted.

**Results:**

A total of 277 patients with hypospadias repair were investigated. The mean age was 3.7+/- 3.5 years (Range, 0.5–14 years) and only one-third (98,35.4%%) of patients were operated on in the recommended age group (6–18 months). Anterior/distal hypospadias was the commonest (123,44.4%) variant identified. The majority (176,63.5%) had chordee and 105(37.9%) were severe forms. Tubularized incised plate repair was the major (164,59.2%) surgical technique employed followed by staged urethroplasty (61,22%). Post-operative complications occurred in 135(48.7%) patients and the commonest was urethrocutanous fistula (95,34.3%). No significant correlation was found between the occurrence of these complications and factors such as age at repair, the severity of hypospadias, presence of concomitant urogenital anomaly, type of procedure and duration of urinary diversion. However, the presence of severe chordee (AOR=3.09; 95%CI 1.21–7.54; p=0.013) was an independent factor found to be associated with postoperative complications on multivariate analysis.

**Conclusion:**

Higher rate of complications following hypospadias repair was observed in our study. Our study also demonstrated no significant advantage of any repair technique in reducing operative complications. Extensive preoperative evaluation, proper operative plan and regular follow-up of such patients is paramount for a better outcome.

## Introduction

Hypospadias is a urogenital congenital anomaly involving the ventral side of the penis characterized by an ectopic ventral opening of the urethral meatus with or without ventral curvature of the penis(chordee) and defective ventral distribution of the foreskin (1–7). It is the second most common congenital anomaly in newborn males after undescended testis with an incidence of 1 in 300 and varies between 0.4–8.2 per 1,000 newborns (1,4–7). Recently, the incidence is rising worldwide due to increasing environmental pollution (1,4).

Hypospadias occurs due to a developmental arrest of the male urethra during the 8th to 16th week of intrauterine life. In the majority of the cases, the etiology is unknown, however, hormonal Interferences in the androgen metabolism and genetic predisposition are incriminated in the pathogenesis (1,4,6,8). Hypospadias may occur as a single anomaly or associated with other congenital urogenital anomalies such as undescended testis (UDT), inguinal hernia, pelviureteric junction (PUJ) obstruction, vesicoureteral reflux, and renal agenesis (8,9).

The abnormal opening of the urethra can be located anywhere from the glans of the penis to the perineum (2 4,8,10). In the majority (70%) of the boys, the meatus lies distally on the penile shaft and represents mild form whereas, 30 % has proximal meatus and are severe forms (6,11).

Surgical correction of the anatomic defect is the mainstay of treatment. Literature described over 300 hypospadias repair techniques (4,9,13). The Tubularized incised plate (TIP) urethroplasty (Snodgrass technique) is preferred by most surgeons for the repair of anterior/distal hypospadias however, more complex reconstruction techniques(two-staged) are used in the management of posterior/proximal hypospadias (4).

If hypospadias is not managed appropriately, it will affect the patient's psychological, emotional and sexual well-being. The success is judged by its cosmetic and functional outcome. Overall, the outcome of hypospadias repair depends on factors such as age at repair, the type of hypospadias, severity of chordee, associated urogenital anomalies, surgical technique used, duration of urinary diversion(stenting), availability of microsurgical instruments and experienced surgeon (2,12,14).

Complications can occur following any form of hypospadias repair surgery. Bleeding, edema, urinary retention, wound infection, wound dehiscence, skin and flap necrosis are the major early complications of hypospadias repair. On the other hand, meatal stenosis, urethrocutanous fistula, urethral diverticula, recurrent chordee, urethral stricture and hairy urethra are among the late complications (3,7,8,15–19).

Despite the huge burden of the condition, data are scarce in our country. So far there are only two published literatures on hypospadias repair. Therefore, this study aims to assess the clinical profiles and surgical outcome (complications) of hypospadias repair.

## Patients and Material

Between September 1^st^, 2015 and August 31^st^, 2019, a retrospective chart review of all pediatric patients (<14 years) who undergone hypospadias repair was done to assess the surgical outcome and factors associated with postoperative complications. The study was conducted at SPHMMC which is a tertiary teaching hospital found in Addis Ababa, Ethiopia. The Pediatric surgical unit provides both inpatient (18 beds) and outpatient services for children referred from all over the country. Two pediatric surgeons and final-year residents under supervision were involved in the care of the patients.

Patients' medical record numbers were identified from the operation theater registry and charts were retrieved from the archive room. A total of 308 pediatric patients underwent hypospadias repair in the study period. Of these, 277(89.9%) patients had complete documentation of at least 6 months follow-up and were included in the study. Data on sociodemographic characteristics, clinical presentation, operative technique and surgical outcome (postoperative complications) was collected from the patient's chart using a pretested data collection format.

The data were checked for completeness and consistency then entered, coded and analyzed using SPSS version 23. Factors associated with postoperative complications were identified using binary logistic regression. An independent variable with a p-value < 0.2 during a bivariate analysis was further subjected to multivariate analysis and considered significant at p-values < 0.05. Texts, central tendency statistics and tables were used to present the findings of the study,

The study was carried out after approval was obtained from SPHMMC institutional review board (IRB). The IRB waived the study without patients' consent due to the retrospective nature of the study. Patients were not contacted and data was entirely collected from the patient's chart. Throughout the study, all data were anonymized and kept confidential.

## Results

A total of 308 pediatric patients were operated on for hypospadias. Of these, 277(89.9%) cases were investigated. The mean age was 3.8+/- 3.5 years (Range,0.5-14 years) and more than half (152,54.9%) of the patients reside in a rural area. The majority (179,64.6%) of the boys were above the age of 1.5 years (18 months). Of these, nearly two-thirds (114, 63.7%) came from outside the capital city, Addis Ababa.

Most (123,44.4%) of the boys were presented with an anterior type of hypospadias. Among the specific types, coronal (68, 24.5%), glandular (55,19.9%) and penoscrotal (46,16.6%) were the top three (Table 1). Among the patients who were operated on after the age of 18 months, 89(49.7%) had an anterior type of hypospadias, 62(22.4%) had middle and 28(15.6%) had a posterior type.

**Table 1:** Types of hypospadias in patients operated for hypospadias at St. Paul's hospital millennium medical college, Addis Ababa, Ethiopia, from September 2015 to August 2019

General types	Specific types	Total n (%)
Anterior	Glandular	55(19.9%)
	Coronal	68(24.5%)
Middle	Distal penile	41(14.8%)
	Midshaft	9(3.2%)
	Proximal penile	39(14.1%)
Posterior	Penoscrotal	46(16.6%)
	Scrotal	5(1.8%)
	Perineal	14(5.1%)

The majority (176,63.5%) of the hypospadias had chordee. Of these, 105(59.7%) were severe and 71(40.3%) were mild forms. Moreover, chordees of the posterior hypospadias (61,98.4%) (p<.0001) were tend to be more severe than anterior (4,8.7%) and middle (40,61.5%). More than two-thirds (242,87.3%) of patients had incomplete prepuce.

Associated urogenital anomalies were noted in 99(35.7%) patients and 17(17.2%) of these had more than one anomaly. The occurrence of associated anomalies increases (p<.0001) as the hypospadias become proximal; anterior (24,19.5%), middle (26,29.2%) and posterior (49,75.4%). Microphallus (29,10.5%) was the commonest anomaly followed by penile rotation (21,7.6%) and undescended testis (18,6.5%). Of the patients, 8(2.9%) had a disorder of sex development (DSD).

The majority (205,74%) of hypospadias repairs were performed in one stage approach. The three most frequently performed surgical techniques were tabularized incised plate (TIP) repair (168,60.6%), staged urethroplasty (72 ,26%) and Mathieu's repair (26,9.4%). The most common procedure employed for each type of hypospadias include TIP for anterior (95,77.2%) and middle (65,73.0%) hypospadias and staged urethroplasty for posterior hypospadias (57,87.7%) (Table 3). Polyglactin (Vicryl) 5/0 was the type of suture material used for both the urethra and glans. All patients received antibiotics either orally or intravenously. Transurethral urine diversion with a Pediatric feeding tube (4–6 FR) was done on all patients and mean time of removal was 7.8+/-4.01 days (Range,1–20 days). Neither magnification nor microsurgical instruments were used in our patients.

**Table 3:** Surgical techniques performed in patients operated for hypospadias at St. Paul's hospital millennium medical college, Addis Ababa, Ethiopia, from September, 2015 to August, 2019

Surgical Technique	Hypospadias Type			
		
	Anterior n (%)	Middle n (%)	Posterior n (%)	Total
Tabularized incised plate	95(77.2%)	65(73.0%)	8(12.3%)	168(60.6%)
Staged urethroplasty	0(0%)	15(16.8%)	57(87.7%)	72(26.0%)
Mathieu's repair	26(21.1%)	0(0%)	0(0%)	26(9.4%)
Onlay island flap	0(0%)	5(5.6%)	0(0%)	5(1.8%)
MAGPI*	3(2.4%)	0(0%)	0(0%)	3(1.1%)
Island flap	0(0%)	3(3.4%)	0(0%)	3(1.1%)

* MAGPI, Meatal Advancement and Glanuloplasty Incorporated

Nearly half (135,48.7%) of the patients developed postoperative complications and no death was recorded. Of these, 8(5.9%) patients had more than one complication. Among patients with anterior, middle and posterior types of hypospadias, complications occurred in 40 (32%), 47(52.8%) and 48(73.8%) cases, respectively. Urethrocutanous fistula (95,34.3%) and Meatal stenosis (24,8.7%) were the two common postoperative complications (Table 4).

**Table 4:** Postoperative complications in patients operated for hypospadias at St. Paul's hospital millennium medical college, Addis Ababa, Ethiopia, from September, 2015 to August, 2019.

Postoperative complications	Total n (%)
No complication	142(51.3%)
Urethrocutanous fistula	88(31.8%)
Meatal stenosis	21(7.6%)
Urethral stricture	7(2.5%)
Urinary tract infection	4(1.4%)
Urethrocutanous fistula and recurrent chordee	3(1.1%)
Glans breakdown	2(0.7%)
Wound Infection	2(0.7%)
Bleeding	2(0.7%)
Urethrocutanous Fistula and stricture	2(0.7%)
Urethrocutanous Fistula and meatal Stenosis	2(0.7%)
Recurrent chordee	1(0.4%)
Recurrent chordee and meatal stenosis	1(0.4%)

**Factors associated with postoperative complications**: On bivariate analysis, the type of hypospadias, presence of chordee, concomitant urogenital anomaly, type of procedure and duration of the catheter were significantly associated with postoperative complications. However, the presence of severe chordee (AOR=3.09; 95%CI 1.21–7.54; p=0.013) remained significantly associated with postoperative complications on multivariate analysis (Table 5).

**Table 5:** Factor associated with postoperative complications in patients operated for hypospadias at St. Paul's hospital millennium medical college, Addis Ababa, Ethiopia, from September, 2015 to August, 2019

Variables	Postoperative complications		Crude odds ratio (95%CI)	Adjusted odds ratio(95%CI)

	Yes	No		
**Age**				
<18 months	51	41	1	1
>=18 months	84	101	0.67(0.40–1.10)	1.02(0.57–1.82)
**Type of Hypospadias**				
Anterior	40	83	1	1
Middle	47	42	2.43(1.38–4.26) **	2.57(0.89–7.42)
Posterior	48	17	5.86(2.99–11.44) **	2.63(0.59–11.69)
**Chordee**				
No chordee	31	70	1	1
Mild to moderate	30	41	1.75(0.93–3.30)	1.47(0.74–2.92)
Severe	74	31	5.39(2.29–9.78) **	3.09(1.21–7.54) *
**Urogenital anomaly**				
Yes	58	41	1.81(1.10–2.98) *	0.96(0.51–1.80)
No	77	101	1	1
**Surgical Technique**				
Mathew's	7	19	1	1
TIP	67	101	1.80(0.72–4.52)	1.23(0.45–3.35)
Staged	55	17	8.78(3.16–24.43) **	3.38(0.80–14.13)
Island Flap	2	1	5.43(0.42–69.76)	2.92(0.19–45.63)
On lay island flap	3	2	4.07(.59–29.73)	1.79(0.19–16.20)
MAGPI	1	2	1.36(.10–17.42)	1.32(0.10–17.71)
**Catheter duration**				
≤ 1 week	50	87	1	1
>1 week	85	55	2.6(1.60–4.23) **	0.39(0.13–1.18)

* Significantly associated at p-Value<0.05

** significantly associated at p-value<0.005

## Discussion

Hypospadias is among congenital anomalies which require surgical correction early in life. Considering the risk of anesthesia, tissue dimension, postoperative outcome, patients' choice and psychological impact, the recommended age for hypospadias repair is between 6 months and 18 months. During this period the child has no genital awareness, not yet started toilet training and ambulation. Furthermore, perioperative management like urinary drainage, hospitalization and separation anxiety are easier in this age group (1,2,6,8,9,20–22). Literature also reported positive body image and improved psychosexual outcomes in patients operated early in life (1,2,6,23). This review and similar studies done in Ethiopia and other developing countries demonstrated late presentation and higher mean age (older than 2 years) at the time of hypospadias repair (8,9,16,17,20,22). In contrast, studies from developed nations reported early surgical intervention (2,24). This may reflect a lack of access to essential surgical services, low public awareness and financial problems (8,9). Our review (65%) and a report by Mammo TN et al (80%) identified a higher burden of cases outside the capital city which might suggest limited service of hypospadias treatment at the periphery which in turn contribute to the late presentation (12).

Based on Duckett's classification, the anterior/distal type of hypospadias was the commonest form in our study. This also holds true in reports from Ethiopian, African and western authors (2,4,12,13,16,17,20,24,25). In contrast, Baskin LS et al and Subramaniam R et al reported posterior/proximal type as the commonest form observed in Asia (5,10). Furthermore, an Indonesian study demonstrated a similar finding. Studies done in Nigeria and Pakistan found the middle type as the dominant form (9,18,22). This discrepancy could be explained by differences in study subjects and setup.

Apart from the ectopic opening of the urethral meatus, hypospadias may occur with or without chordee/ventral penile curvature. It is resulted from abnormal development and fusion of various levels of the ventral tissues of the penis such as the skin, Buck's fascia, corpus spongiosum and tunica albuginea (1,20). In this study, nearly two-thirds (63.5%) of hypospadias had some degree of chordee which is in line with the reported range (25%–88%) (9,12,14,17,20). However, the occurrence of a severe form of chordee is higher in our patients (37%) than studies done in Ethiopia (31%), Tanzania (20%), Nigeria (29.9%) and Pakistan (7.5%) (9,12,16,22). The lowest rate in the latter study could be due to the involvement of adult patients in their study, hence patients with mild disease tend to seek medical attention late.

Various forms of urogenital anomalies such as penile rotation, microphallus, penoscrotal transposition, ambiguous genitalia, undescended testis, inguinal hernia, pelviureteric junction (PUJ) obstruction, vesicoureteral reflux and renal agenesis can occur in combination with hypospadias (1,4,8,9,11). Our review and studies done in Ethiopia and Indonesia found 35.7%,18.3% and 28.6% associated urogenital anomalies, respectively (8,12). Mammo TN et al and Schneuer FJ et al noted a greater likelihood of associated urogenital anomalies in proximal defects which is also the case in our study (7,12). UDT is among the commonest associated anomaly identified in this review and studies done elsewhere (1,2,8,9,12,17,25). Aulia l et al reported microphallus as the commonest anomaly (14.5%) which is also true in the present study (10.5%).8 If hypospadias occurs with UDT and small size penis(<2.5cm), intersex anomaly should be suspected and worked up with imaging, hormonal analysis and karyotyping (9,26). A recent Ethiopian study and our study found DSD in 4.5% and 2.9 % of study subjects, respectively (12). PUJ obstruction was the only concomitant upper urinary tract malformation noted in the recent study. Authors also reported vesicoureteral reflux and renal agenesis associated with severe cases of hypospadias (1).

In the first and second centuries, AD Celsius and Galen shared their experience on hypospadias repair since then over 300 repair techniques have been described to improve functional and cosmetic outcomes (3,4,9,10,13,14,19,24). This fact reflects that there is no universally accepted single technique fit for all variants of hypospadias (19,13,26,27). However, all techniques strive to promote urination on standing position without interruption and spraying, to achieve normal coitus and insemination, and appropriate penile cosmesis (3–6,12,14,25). The choice of technique is determined by meatal site, penile curvature and size, the status of the foreskin, quality of urethral plate and availability of experienced expertise and setup (10,11).

Literature reported that the majority of the repairs can be performed in one stage approach. Complex repair techniques (two-staged), on the other hand, are reserved for severe (proximal) forms of hypospadias (1,4,9–11). In line with this, more than two-thirds (74%) of our cases underwent one stage hypospadias repair and two-staged procedures were done for the majority (87.7%) of posterior/proximal defects. A worldwide survey on hypospadias surgery revealed 52.9%–71.0% of surgeons prefer TIP for the repair of distal (anterior and midshaft) defects.28 Similarly, the recent study and reports from other literature found agreeable results (12–14,16,18,20,22). Springer A et reported 0.9–16.7% of surgeons use TIP for proximal hypospadias repair.28 This is consistent with the finding of our review which is 12.3%. On the other hand, a study done in Pakistan and Sudan found two-stage repair (46.4%) and MAGPI (42%) as the choice of hypospadias repair in general, respectively (9,17) Such variation may be explained by difference in the choice and experience of expertise. Khan M et al mentioned a relatively short learning curve for two stage repair and low awareness toward single-stage procedure as the possible reason for the discrepancy.9 In agreement with our finding, studies done locally and elsewhere found Mathieu's repair as among the commonly performed procedure for the treatment of distal hypospadias (13,17,18,20). Even if it is controversial, many experts recommend urinary diversion after hypospadias repair. It is a routine practice to use transurethral urinary diversion in our setup and others. However, the availability of appropriate pediatric urethral Catheter is limited in developing countries, instead a pediatric feeding tube (6–8 FR) is used (12–14,18,20). Long-term use of urinary diversion is not advised unless it is extensive urethroplasty (26). A study done locally at a tertiary care hospital reported 7 days as an average duration of transurethral diversion which is also noted in our review (12).

Sub-optimal management of hypospadias affects patients' psychological, emotional and sexual well-being. Despite the evolution of various repair techniques, operative complications of hypospadias remain a challenge and could occur following any form of reconstructive surgery (5,13,17). However, the occurrence of complications could be reduced with proper patient evaluation and application of the surgical principles of hypospadias repair. These principles include tension free repair, use of loupe magnification and microsurgical instruments, gentle handling of tissue, minimal (precise and brief) use of cautery, use of well-vascularized tissue closure as many layers as possible, keeping tissues moist and rational use of prophylactic antibiotics (3,5,7,13,17,26,29). In addition, patients should be followed regularly in the postoperative period (at 1st week, 1st month, 3rd month, 6th month then yearly for two years) and the final outcome is judged only at mid-teen age or puberty (1,9). In our review, all the patients were followed for at least 6 months.

Literature reported varying rate of post-operative complications ranging between 6 and 68% (2,3,9,11–17,20,26,27,30). Even if the complication rate in our review (48.7%) is within the reported incidence, it is relatively higher than most studies. Local studies done by Hagos M (13.4%) and Mammo TN et al (44.1%) found a lower rate of complications than ours (12,20). This may be due to differences in patients' characteristics and duration of postoperative follow-up. Moreover, the disparity in surgery and training practice may also attribute to the variation (2). Almost all centers reported UFC as the commonest complication following hypospadias repair(range,7.7–47%) which is also demonstrated in the present study (34.3%) (2,3,9,11–17,20,26,27,30). Furthermore, results from the current study (8.7%) and others (3–14%) showed meatal stenosis as the second most common complication of hypospadias surgery (2,3,9,11,16,19,30) Other complications such as infection and persistent/recurrent chordee are decreasing due to judicious use of prophylactic antibiotics and erection test during the procedure (26,29). By following the operative principles of hypospadias repair, Bhat A et al reported a reduction of post-operative complications (in plate preservation procedures) to < 5% and < 10% in distal and proximal hypospadias, respectively (3).

**Factors associated with postoperative complications**: Authors mentioned factors such as age at repair, type of hypospadias, the severity of chordee, associated urogenital anomalies, the complexity of surgical technique, use and duration of urinary diversion, availability of microsurgical instruments and experience of the operating surgeon as outcome predictors of hypospadias repair (2,3,12,14–16,19). The Multivariate analysis of our review found the presence of severe chordee as the only factor significantly associated with increased odds of postoperative complication (AOR=3.09; 95%CI 1.21–7.54; p=0.013) than their respective groups. This is in line with similar studies done locally and elsewhere (12,14,16). Mammo TN et al found 59.7% and 34% rates of complications in those with severe and no chordee, respectively (12). On the other hand, the current study and results from other literature found no statistically significant correlation between complications of hypospadias surgery and factors like age at repair, presence of concomitant urogenital anomalies and surgical technique performed (2,7,11,31). In contrary to this, other similar studies done in Africa, western and globally demonstrated a significant effect of these factors in the occurrence of postoperative complications (2,3,12,16,18,21,26). This discrepancy might partly be explained by differences in study subjects and the method of analysis used. Duration of urinary diversion has no correlation with a higher rate of an unfavorable outcome as it is noted in our study and others (12,14,32).

This study provided important data to our setup which helps in the management of such a common and challenging condition, hypospadias. However, it is not without limitation. Short postoperative follow-up, the absence of data regarding details of the techniques and experience of the operating surgeon are the major drawbacks which are mainly attributed by the retrospective nature of the study.

In general, most of our patients were operated later than the recommended age. TIP and two-stage procedures were the technique of choice in distal/middle and proximal hypospadias, respectively. A higher rate of operative complications was observed in our setup than in most centers. The presence of severe chordee was the only factor significantly associated with increased odds of operative complications. As far as these complications are concerned, our study also demonstrated no significant advantage of any repair technique. Extensive evaluation, proper operative plan and regular follow-up is paramount in the management of hypospadias, particularly for those with severe chordee. prospective studies with long term follow-up need to be conducted for further results.

## Figures and Tables

**Table 2:** Associated urogenital anomalies in patients operated for hypospadias at St. Paul's hospital millennium medical college, Addis Ababa, Ethiopia, from September, 2015 to August, 2019

Urogenital Anomaly	Total n (%)
No anomalies	178(64.3%
Microphallus	17(6.1%)
Penile rotation	16(5.8%)
Inguinal hernia	14(5.1%)
Undescended testis	12(4.3%)
Pelvi-uretral junction obstruction	10(3.6%)
Disorder of sex development	8(2.9%)
Penoscrotal transposition	5(1.8%)
Penile rotation and microphallus	5(1.8%)
Penoscrotal transposition and microphallus	4(1.4%)
Penoscrotal transposition and Undescended testis	4(1.4%)
Undescended Testis and microphallus	2(0.7%)
Penoscrotal transposition and Pelvi-uretral junction obstruction	1(0.4%)
Inguinal hernia and microphallus	1(0.4%)
